# Combination of Hypofractionated Radiation Therapy and Gilvetmab for the Treatment of Cutaneous or Subcutaneous Mast Cell Tumours in Dogs

**DOI:** 10.1111/vco.70110

**Published:** 2026-09-01

**Authors:** Tiffany W. Martin, Douglas H. Thamm, Kristen M. Weishaar

**Affiliations:** ^1^ Department of Clinical Sciences, College of Veterinary Medicine and Biomedical Sciences Colorado State University Fort Collins Colorado USA

**Keywords:** canine, immunotherapy, toxicity

## Abstract

Gilvetmab is a caninised monoclonal antibody against canine PD‐1. Concurrent or sequential administration of anti‐PD‐1 antibodies and radiation therapy (RT) improves survival time in various species. Eighteen dogs with mast cell tumours (MCTs) were prospectively treated with 24 Gy of RT over Days 0, 7 and 21 and gilvetmab every 2 weeks beginning on the second fraction of RT (Day 7) for up to 10 doses. Best tumour response was complete in one (6%), partial in eight (44%) and stable disease in nine dogs (50%) for an ORR of 50%. Median time to best response was 7 days after the first fraction of RT. Thirteen dogs (72%) developed progressive disease with a median progression free interval (PFI) of 116 days after the first fraction of RT. Nine adverse events (AEs) attributed to gilvetmab were recorded in seven dogs (39%) consisting of infusion reactions in four, metabolic changes in three and gastrointestinal signs and pancreatitis in one each. Overall, five dogs (28%) experienced Grade 3 AEs. Radiation AEs were limited to Grade 1 for both acute and late effects in seven dogs (39%). There was a significant difference in PFI and MST for dogs with and without lymph node metastasis with a median PFI of 64 versus 292 days and MST of 103 versus 587 days (*p* = 0.02 and 0.005, respectively). This sequence, or combination, of therapies did not provide durable tumour control for canine MCT and the AE profile warrants further evaluation.

## 1 Introduction

Ionising radiation therapy (RT) has been shown to upregulate programmed death‐ligand 1 (PD‐L1) in tumour cells [1, 2]. Concurrent or sequential administration of anti‐PD‐1 antibodies and RT has been shown to improve outcomes in mice, people and dogs [3, 4, 5]. In a colonic carcinoma mouse model, a significant improvement in overall survival was seen in mice administered PD‐1 blockade on Day 1 or Day 5, but not those given the blockade on Day 7 (which was considered sequential) [6].

Mast cell tumours (MCTs) are common cutaneous tumours in dogs and, when not amenable to surgical excision, can present a therapeutic challenge. Radiation therapy is an effective option for non‐resectable tumours, but its greatest benefit is observed in the microscopic disease setting [7]. New treatment protocols for unresectable MCT are needed. Gilvetmab is a caninised monoclonal antibody against canine PD‐1 that has been assessed in dogs with macroscopic MCT. The objective response rate alone is reportedly 46%, with most dogs with macroscopic MCT experiencing a partial response (PR) [8]. Gilvetmab appears to be well tolerated, with only five Grade 3–5 adverse reactions reported from 26 dogs, resulting in a total of three dogs being withdrawn from the study [8]. The combination of gilvetmab and hypofractionated RT has not yet been evaluated for MCT in dogs.

The objectives of this prospective study were (1) to determine the overall response rate (ORR; complete response and partial response), (2) evaluate the time to best response and duration of response and (3) evaluate adverse effects associated with combined therapy of gilvetmab with hypofractionated RT. Our hypothesis was that dogs receiving combination hypofractionated RT and gilvetmab would experience an ORR of 70% or greater with fewer than 15% of patients experiencing Grade 3–5 adverse reactions.

## 2 Materials and Methods

### 2.1 Case Recruitment

Patients for this prospective clinical trial were recruited from dogs presenting to the Flint Animal Cancer Centre at Colorado State University between May 2024 and June 2025 for evaluation of measurable MCT. Owners were presented with standard of care surgical options or RT outside of the clinical trial. All owners gave informed consent for their dogs to participate in this study and all methods were approved by the University Clinical Review Board responsible for assessing animal usage (#5430). Dogs with previous RT to the primary tumour or those with disseminated visceral metastatic disease were excluded from enrolment. Dogs with focal visceral metastasis were eligible to enrol. Additionally, dogs that could not undergo general anaesthesia for any reason or those that received chemotherapy in the previous 3 weeks were ineligible for enrolment.

All dogs were required to have a cytologically or histologically confirmed diagnosis of cutaneous or subcutaneous MCT with measurable disease present that was considered amenable to hypofractionated RT. Locoregional lymph node aspirates were performed prior to enrolment (if feasible). Dogs with lymph node metastasis were eligible to enrol. Thoracic and abdominal imaging were required within 1 month of starting the trial. This could be via thoracic radiographs or computed tomography (CT) and abdominal ultrasound or CT. CT was performed with a Siemens Somaton Force 128‐slice scanner (Siemens Medical Solutions, Malvern, Pennsylvania). Any concerning areas on imaging required sampling via fine needle aspirate and cytology. Additionally, all dogs were required to have adequate organ function based on complete blood count (CBC) and biochemistry profile including an absolute neutrophil count of > 2000/μL, haematocrit > 30%, platelets > 75 000/μL, creatinine < 2 times the upper normal limit, bilirubin ≤ 0.2 mg/dL and serum transaminases ≤ 3 times the upper normal limit. Dogs were grouped into high and low neutrophil to lymphocyte ratio (NLR) categories based on the median value for dogs in this study. Dogs presenting on an immunosuppressive dose of corticosteroids were required to have a reduction of their dose at least 7 days prior to administration of gilvetmab. All dogs were placed on an anti‐inflammatory dose of prednisone (0.5 mg/kg PO daily), omeprazole (0.7 mg/kg PO daily) and cetirizine (1 mg/kg PO daily) at initial presentation if not already started.

Adverse radiation effects were graded according to the Veterinary Radiation Therapy Oncology Group Toxicity Criteria version 2 (VRTOG v2) with acute effects occurring within the first 90 days and late effects occurring after 90 days [9]. Dogs were also assessed for toxicity using the Veterinary Cooperative Oncology Group—Common Terminology Criteria for Adverse Events version 2 (VCOG‐CTCAE v2) [10]. Adverse events were recorded at each presentation to the hospital.

### 2.2 Radiation Therapy

The prescribed radiation protocol was 24 Gy in three fractions delivered on Days 0, 7 and 21. Plans were created via intensity modulated radiation therapy (IMRT) or manually calculated plans. For IMRT plans, Eclipse version 15 was utilised for treatment planning with a heterogeneity correction utilising two partial arcs for volumetric modulated arc therapy (VMAT). Beams were modulated using the sliding‐window technique with the goal of delivering 100% of the prescription to 99% of the GTV and 95% of the PTV with the Acuros algorithm.

Patients were treated with either 6 or 10 MV photons or 6, 9 or 15 MeV electrons depending on the mass and clinician discretion. A 5 mm, 8 mm or 1 cm bolus was used in all patients except one. Manually calculated plans utilising photons were delivered with parallel opposed fields and a fixed source to axis distance (SAD) while manually calculated electron plans were delivered with a single field with a source to skin distance (SSD) technique. The calculation point for photons was the isocentre and for electrons was the 95%–100% isodose line of the specific electron energy. For manually calculated photon plans, a megavoltage (MV) portal image was captured and evaluated prior to treatment each day to ensure appropriate patient positioning. For IMRT plans, a kilovoltage cone beam computed tomography (kv‐CBCT) scan was performed prior to treatment to assure appropriate positioning. For both manually calculated plans and IMRT, the palpable tumour or tumour identified on CT was identified as the gross tumour volume (GTV). A 1 cm planning target volume (PTV) was used for manually calculated plans while a 5 mm PTV was used for IMRT plans. No clinical target volume was used in any case.

A quality assurance (QA) plan was created for IMRT plans and measured via gamma analysis using the Varian portal dosimetry system on each arc prior to treatment. A minimum of 95% gamma at a 3 mm distance to agreement and a 3% absolute dose difference was required to pass.

All patients were anaesthetised for their RT. While the anaesthetic protocol varied for each patient, most included no pre‐medication drugs and induction with diazepam and propofol. After intubation, patients were maintained on an isoflurane and oxygen admixture. Patients were positioned so that their tumour was as isolated as possible for treatment for manual calculations. For IMRT, lateral recumbency was utilised in a mouldable bag (Vac‐Lock Cushions; CIVCO Medical Solutions, Coralville, IA). The mouldable bag was indexed to the CT and radiation therapy couches. Therapy was delivered using a Varian Trilogy linear accelerator.

### 2.3 Gilvetmab

Patients were administered 10 mg/kg of gilvetmab IV as an infusion over 30 min every 2 weeks beginning on the day of the second fraction of RT (Day 7) for up to 10 doses, as long as their disease was assessed as stable or better.

### 2.4 Follow up

The longest tumour diameter was recorded at initial presentation (Day 0), prior to RT. At each subsequent recheck timepoint and administration of gilvetmab, tumour measurements were performed to assess for response consistent with veterinary response evaluation criteria for solid tumours (RECIST) criteria [11]. The best RECIST response and duration of response were recorded. Dogs were required to maintain stable disease (SD) for at least 3 weeks for this designation. After completion of the 10 doses of gilvetmab, follow‐up visits occurred every 2 months for tumour measurement until the patient was a year out from RT or until disease progression. At that point, visits were scheduled every 4 months.

Dogs were withdrawn from the trial if there was progression of disease, if there were medically significant Grade 3–5 adverse effects or any condition where immediate treatment was required outside of the trial, or if hospitalisation was required due to anaesthetic or drug complications.

### 2.5 Statistical Analysis

A Simon's two‐stage design was used for power calculation [12]. The null hypothesis, that the true response rate is 40% was tested against a one‐sided alternative. A total of 18 patients were recruited and it was determined that the null hypothesis would be rejected if 11 or more responses were observed in 18 patients. This design yielded a Type I error rate of 0.0495 and power of 0.8226 when the true response rate was 70%.

Patients were included on an intent to treat basis. Progression free survival (PFS) was calculated as the time between the first day of treatment and progressive disease (PD) or death. Progression free interval (PFI) was calculated as the time between the first day of treatment and PD. Dogs were censored from progression at the date of analysis if they maintained a response to treatment. Survival time was calculated as the time between the first day of treatment and death. Dogs were censored from survival at the date of analysis if they were still alive or at the date when they were lost to follow up. Kaplan–Meier survival curves were created and overall median survival time (MST), PFI and PFS determined. Log‐rank testing was used to compare survival curves from different variables. Fisher's exact test was used to compare categorical groups, and a Cox regression analysis was used to compare continuous variables and PFI or survival time. A *p* value of < 0.05 was considered statistically significant. All statistical tests were performed with Prism v10.4.1 (Graphpad, San Diego, CA).

## 3 Results

A total of 18 patients were enrolled in the study. Patient characteristics can be found in Table 1. Breeds included five American Pitbull Terriers or Pitbull mixes, two Labrador Retriever or Labrador mixes, two Cattle Dog mixes and one each of the following: Japanese Chin, Bull Mastiff, Shih Tsu mix, German Shepherd mix, Bernese Mountain Dog, Swiss Mountain Dog, Border Collie mix, French Bulldog and Dachshund. The diagnosis of MCT was based on cytology in all cases. Clinical signs related to the MCT included lameness in three, scooting the hind end in one, pawing at the face in one and a suspected degranulation event in one. The remaining 12 dogs had evidence of a mass with no other clinical signs. The MCT was located on a hindlimb in six, the head in two, forelimb in two, tail base in two, thorax in two, perianal in one, cervical region in one, medial retropharyngeal lymph node in one and at the level of a hindlimb amputation site in one.

**TABLE 1:** Patient demographics.

Characteristics	*N*	%
Median age, years (range)	9 (2–13)	
Median weight, kg (range)	28.5 (2–59.8)	
Sex		
Castrated males	11	61%
Intact males	2	11%
Spayed females	4	22%
Intact females	2	6%
Time between diagnosis and enrolment, months (range)	1 (0–6.2)	
Prior surgery	7	39%
Prior chemotherapy*	5	28%
Lymph node metastasis	8	53%

*Note:* Characteristics of dogs included in the study. *One dog received two doses of vinblastine without previous surgery prior to developing progressive disease. At that time, was prescribed toceranib phosphate prior to presenting for enrolment in the clinical trial.

### 3.1 Staging

Fine needle aspirate and cytology were performed for locoregional lymph nodes in 15 dogs (83%). Of those 15, 8 (53%) were cytologically consistent with metastatic MCT, 4 (26%) were reactive or had no abnormalities, 2 (13%) were consistent with mastocytosis and in 1 (7%) the lymph node was the target lesion.

All patients had three‐view thoracic radiographs at enrolment. Fourteen (78%) had normal thoracic radiographs with no abnormalities. Two (11%) had evidence of mild left atrial enlargement and two (11%) had evidence of intervertebral disc disease and disc space narrowing. Seventeen (94%) had an abdominal ultrasound and one (6%) had an abdominal CT for staging for enrolment. Four dogs (22%) had evidence of lymphadenopathy, three dogs (17%) each had evidence of splenic or liver changes, two dogs (11%) had both splenic and liver changes, two (11%) had lymphadenopathy and splenic changes and two (11%) had hepatosplenic changes and lymphadenopathy. One dog (6%) had an adrenal mass with caudal vena cava invasion with lymphadenopathy and one dog (6%) had no abnormalities on abdominal ultrasound. Fine needle aspirate of the spleen was performed in 11 dogs (61%) and cytology was consistent with extramedullary haematopoiesis in five (50%) and normal in five (50%). One dog had cytology consistent with low numbers of mast cells with increased atypia and aggregation most compatible with metastatic neoplasia. The liver was sampled via fine needle aspirate in four dogs (22%) and was normal in two (50%), inconclusive due to low cellularity in one (25%) and mild vacuolar hepatopathy in one (25%). No dogs had evidence of hepatic metastasis at the time of enrolment.

All dogs had a CBC and chemistry profile performed for enrolment. The most common relevant abnormality on the CBC was lymphopenia in five dogs (28%) and monocytosis and eosinophilia in four dogs each (22%). The most common abnormality on the chemistry profile was elevated alkaline phosphatase (ALP) in seven dogs (39%) and elevated globulin in four dogs (22%). The median neutrophil‐to‐lymphocyte ratio (NLR) was 5.75 (2.24–49.8). Four dogs (22%) had a urinalysis (UA) at enrolment with hyposthenuria noted in two (50%), a subclinical urinary tract infection in one (25%) and a normal UA in one (25%). Prior to enrolment in the clinical trial, five dogs (28%) had histopathology performed on the MCT that was treated. Three were cutaneous and one was a High‐Grade 3 and two were Low‐Grade 2. Two were subcutaneous and one each was described as low and high grade. At the time of enrolment, the largest median diameter of the target lesion was 4.9 cm (range 1.4–14 cm). Three dogs (17%) had multiple cutaneous MCTs and all MCTs were treated with RT in two of those dogs.

### 3.2 Radiation Therapy

Dogs were scheduled to receive 24 Gy in three fractions on Days 0, 7 and 21. Sixteen dogs (89%) completed the protocol and two dogs (11%) received two fractions prior to PD and withdrawal from the trial. One dog was treated on Days 0, 7 and 28 due to a linear accelerator malfunction and two dogs were treated on Days 0, 8 and 22 due to owner constraints. Five dogs (28%) were treated using electrons and 13 (72%) were treated with photons. The metastatic lymph node was included in the RT plan for all nine dogs with cytologically confirmed metastatic disease. All dogs, except for one, were treated using a manual plan and one was treated with an IMRT plan. The median anaesthesia time for the first, second and third fractions was 30, 15 and 10 min, respectively (range 10–75 min). One dog experienced mild hypotension during the third fraction and one dog experienced a delayed anaesthetic recovery during the first fraction; otherwise, no anaesthetic complications were noted.

### 3.3 Gilvetmab Administration

Five dogs (28%) completed 10 doses of gilvetmab. Ten dogs (56%) were withdrawn from the study due to PD with three dogs prior to Dose 2, two dogs prior to Dose 3, two dogs prior to Dose 4, two dogs prior to dose 10 and one dog prior to Dose 5. One additional dog was withdrawn prior to Dose 7 due to PD and a delay in administration of Dose 7 due to adverse events. One dog was withdrawn from the study after the mass ruptured and was subsequently removed by the primary care veterinarian prior to administration of Dose 3. One owner elected to remove a dog from the trial prior to Dose 6 for non‐medical reasons.

### 3.4 Adverse Events

Throughout the trial period, 16 adverse events (AEs) were recorded in 12 dogs (67%). Adverse events were categorised as infusion reaction in four, metabolic in three and gastrointestinal signs and pancreatitis in one each. RT AEs occurred in seven dogs (39%). Overall, five dogs (28%) experienced Grade 3 AEs. Dogs with a high NLR (greater than the median of 5.75) were not at a higher risk of developing AEs (*p* > 0.99).

All dogs with infusion reactions were attributed as likely due to gilvetmab. One dog had two separate Grade 2 infusion reaction events at Doses 2 and 3 which included vocalising, thrashing and body rigidity prior to becoming lax and cyanotic during the infusion. The event lasted for 5–10 s and resolved without medical intervention. The second episode included dry heaving and vomiting five times with diarrhoea and tenesmus during the final minutes of the infusion. This also resolved within 15–30 min. This patient was pre‐treated with maropitant for all subsequent gilvetmab infusions. Another dog had a Grade 1 infusion reaction during the infusion including lip smacking and panting during Dose 3. The rate of infusion was reduced and the signs resolved without incident. One additional dog had a Grade 3 infusion reaction during the 3rd infusion which included becoming laterally recumbent and seemingly unresponsive. Diphenhydramine was administered and the patient became more alert and stable; however, the remainder of the 3rd dose was not completed resulting in the patient receiving 71% of the intended prescribed dose. Diphenhydramine was administered prior to all subsequent gilvetmab infusions.

Dogs with metabolic AEs were all attributed as possibly due to gilvetmab. These included a Grade 3 elevation in ALP in all three. One of these dogs also had a Grade 3 elevation in alanine aminotransferase (ALT) and gamma‐glutamyl transferase (GGT) with concern for diffuse metastatic disease to the spleen, liver, lymph nodes, peritoneum and body wall on ultrasound. This patient was withdrawn from the trial for PD prior to Dose 2. Another of the dogs with Grade 3 ALP elevation also had a Grade 2 ALT elevation (previously Grade 1 at enrolment), unchanged Grade 1 aspartate aminotransferase (AST) elevation and mild GGT elevation (previously normal) at Dose 4. The final dog with a Grade 3 ALP elevation also had a Grade 2 ALT elevation and mild GGT elevation, which were both previously normal at Dose 3. This patient was clinically asymptomatic.

One dog developed Grade 2 gastrointestinal signs which included 2–3 days of hyporexia, along with lethargy and an increased respiratory rate and effort after Dose 1. The final dog developed Grade 3 pancreatitis 8 days after Dose 6 with vomiting and regurgitation, progressively worsening appetite to complete anorexia, hyperthermia and ultimately required hospitalisation with supportive care for 4 days. Both of these events were considered possibly attributable to gilvetmab.

Radiation AEs were limited to Grade 1 for both acute and late effects. One dog (6%) developed a possible acute Grade 1 skin effect consisting of mild erythema. Six dogs (33%) developed definite Grade 1 late skin effects consisting of hypotrichia or leukotrichia in the RT field.

### 3.5 Follow up and Survival

The best tumour response was complete response (CR) in one (6%), partial response (PR) in eight (44%) and SD in nine (50%). The ORR was 50%. The median time to best response was 7 days (7–36 days) after the first fraction of RT. Figure 1 shows a representative patient with a PR and the progression of response over time. Thirteen dogs (72%) had PD with eight (62%) experiencing local progression of the target lesion and five (38%) experiencing systemic progression outside of the target lesion. The median PFS was 97 days (21–580 days) and the median PFI was 116 days (21–292 days; Figure 2) after the first fraction of RT. There was no significant difference between RECIST groups and development of PD with the one dog with a CR developing progression at 140 days and median PFS of 196 (range 35–580 days) and 49 (21–257 days) days for PR and SD, respectively (*p* = 0.2). There was a significant difference in PFI for dogs with and without lymph node metastasis with a median PFI of 64 days versus 292 days, respectively (*p* = 0.02; Figure 3). The overall MST was 238 days (21–600 days; Figure 4). Dogs diagnosed with lymph node metastasis prior to treatment had a MST of 103 days (range 21–279 days) compared to 587 days (51–600 days) for those without lymph node metastasis (*p* = 0.005).

**FIGURE 1:**
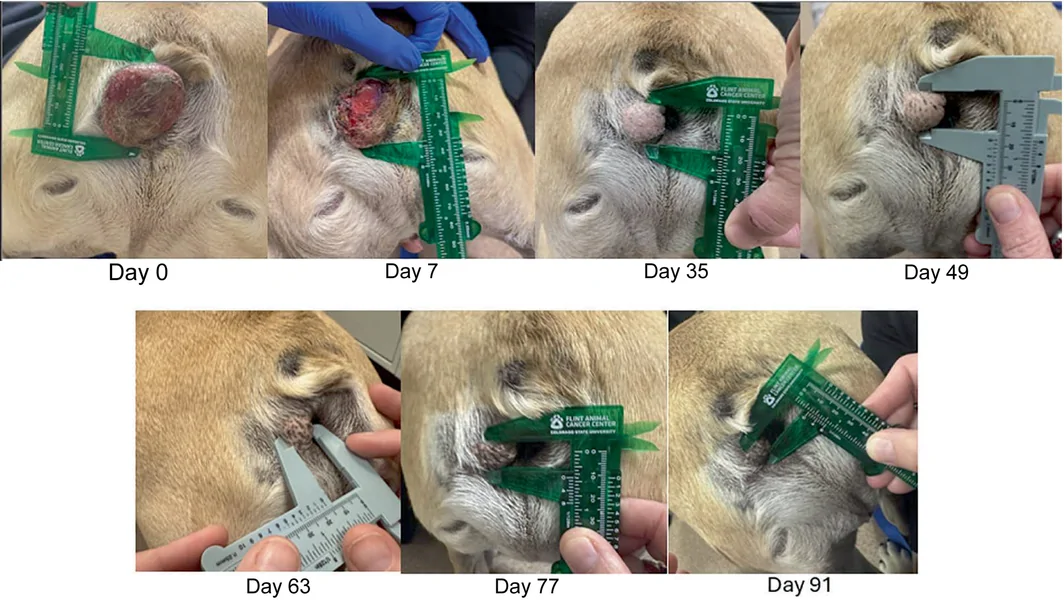
Representative patient with a partial response of a MCT in the perianal region. Partial response was recorded at Day 35 and maintained until 196 days after the start of RT when a new ulcerated MCT appeared ventral to the original target lesion.

**FIGURE 2:**
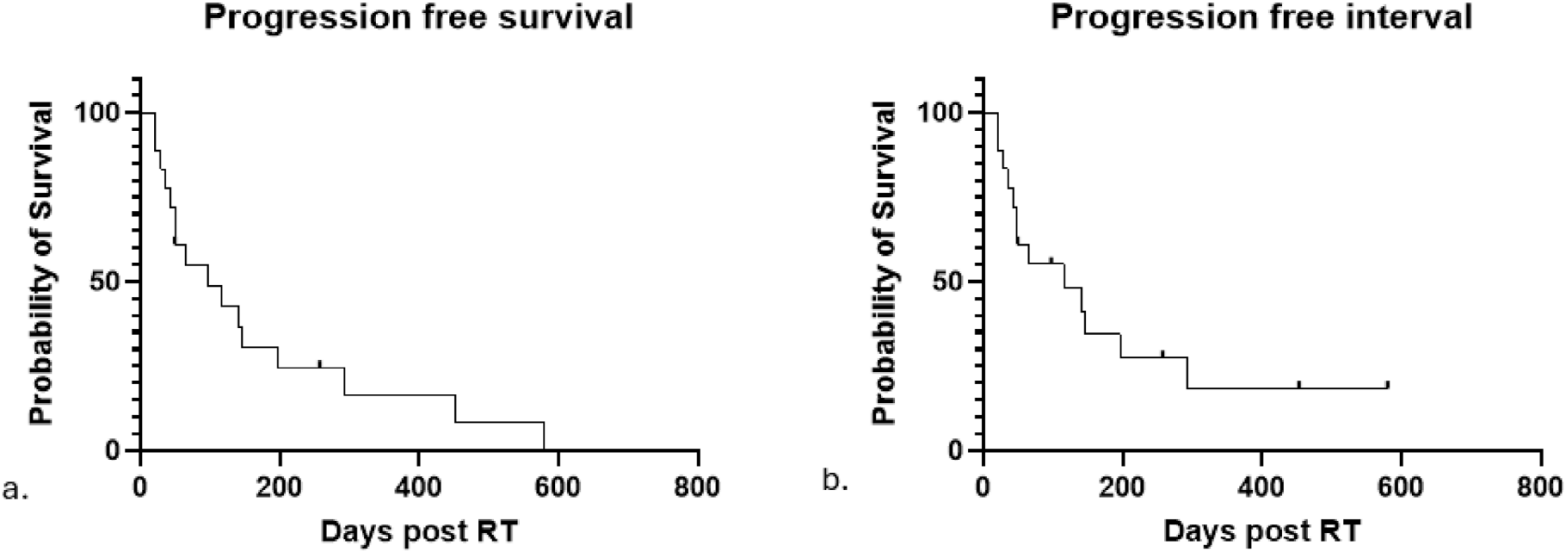
(a) Progression free survival curve depicting a median PFS of 97 days after the first fraction of RT. (b) Median progression free interval was 116 days. Tick marks represent censored patients.

**FIGURE 3:**
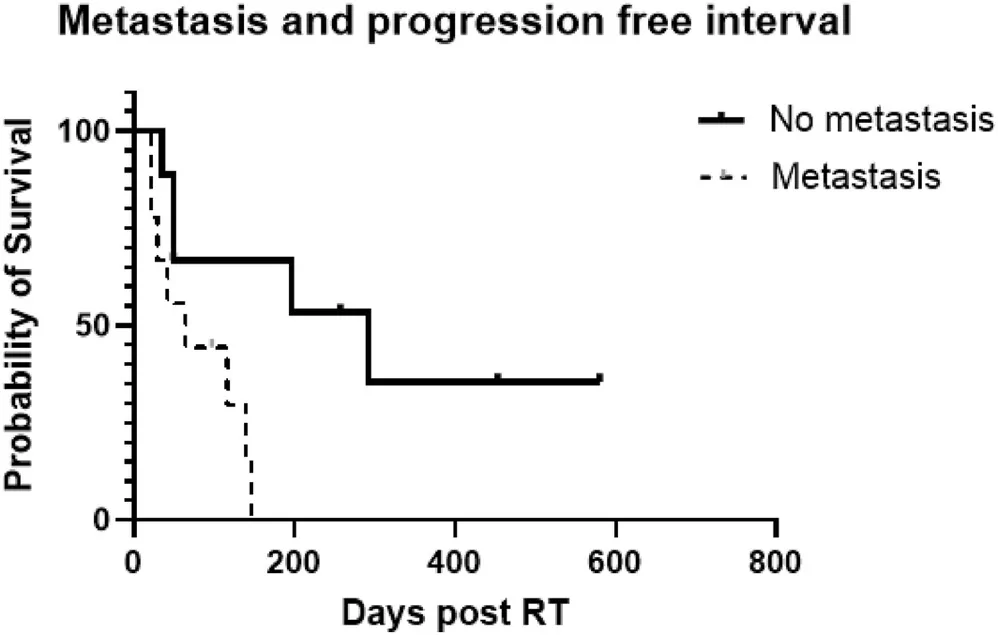
Kaplan–Meier survival curves comparing dogs with and without lymph node metastasis at the time of treatment to PFI. Dogs without lymph node metastasis had a median PFI of 292 days compared to 64 days for dogs with lymph node metastasis (*p* = 0.02). Tick marks represent censored patients.

**FIGURE 4:**
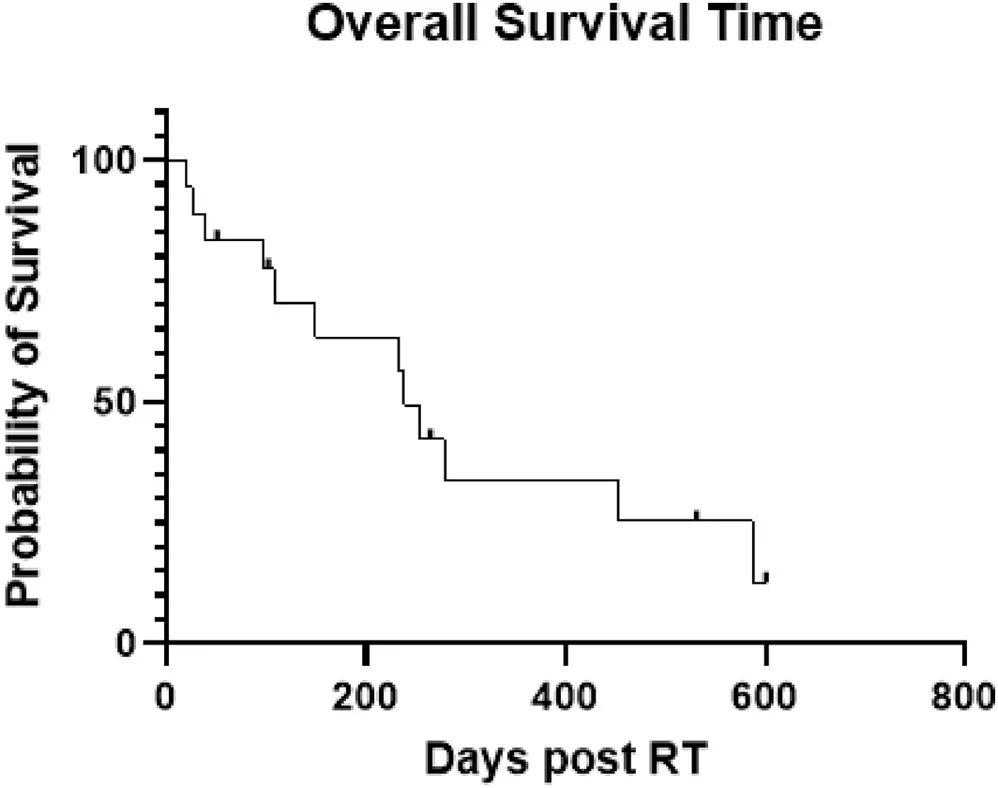
Kaplan–Meier survival curve representing overall survival for all patients. Median survival time was 238 days. Tick marks represent censored patients.

There was a trend toward significance with RECIST groups and dogs in high versus low NLR groups (*p* = 0.057; Figure 5) with dogs in the high NLR group more likely to develop SD. The NLR was not significantly associated with PFI or OST on Cox regression analysis (*p* = 0.64 and 0.12, respectively).

**FIGURE 5:**
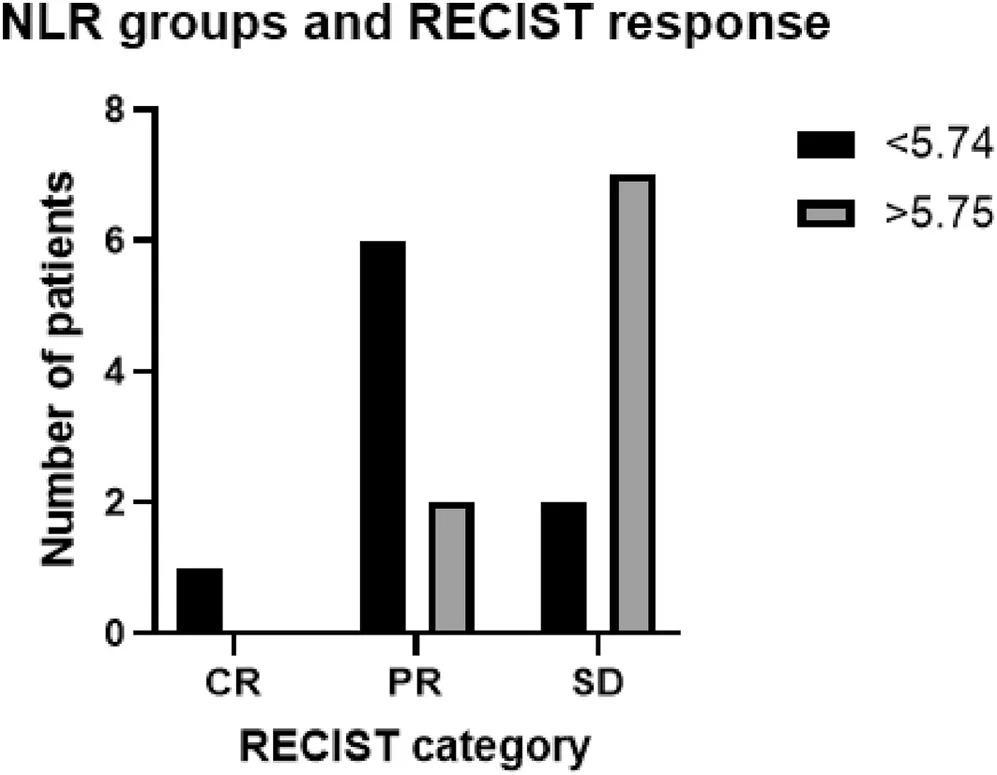
Bar graph depicting patients in either high (> 5.75) versus low (< 5.74) neutrophil to lymphocyte ratio groups and their response with respect to RECIST categorisation (*p* = 0.057).

At the time of analysis, three dogs were still alive, three were lost to follow up after study withdrawal and 12 dogs were euthanised. Cause of death was related to mast cell disease in nine dogs (75%) and unrelated in three dogs (27%).

## 4 Discussion

This study is the first to look at a combination of hypofractionated RT and concurrent gilvetmab for the treatment of MCT in dogs. Our hypothesis was that the ORR would be 70% and the Grade 3–5 AEs would be less than 15%. The data from this study indicate a 50% ORR and Grade 3 AEs in 28% of patients, resulting in a rejection of the hypothesis.

The ORR was lower than hypothesised with a median time to best response of 7 days after the first fraction of RT. This is prior to the first dose of gilvetmab and can only be reflective of the tumour's response to RT. Unfortunately, there are limited studies that evaluate response to hypofractionated RT alone without combination of medical therapy for MCT in dogs [13]. Because of this, it is challenging to determine if the response seen in this cohort of dogs is comparable, better or worse than performing RT alone. Further investigation into response rate, time to best response and duration of response with hypofractionated RT alone is warranted to be used as a monotherapy baseline to compare future combination protocols.

Grade 3–5 AEs with gilvetmab alone are reportedly rare with the most common AE being Grade 1–2 lethargy and fatigue [8]. Here, the most common AE was an allergic reaction or infusion reaction which is not reported by the manufacturer for dogs with MCT; however, it was noted in 6% of normal beagles receiving the infusion [8]. The manufacturer does recommend pre‐medicating with diphenhydramine either orally or intramuscularly 15–30 min prior to each treatment which was not done in this study. Diphenhydramine has poor bioavailability in dogs when administered orally with less than 10% of the administered dose detected systemically [14]. Alternatively, cetirizine has good bioavailability with a plasma terminal half‐life of 10 h [15]. Additionally, when the two drugs were compared, cetirizine outperformed diphenhydramine in preventing cutaneous allergic reactions in dogs [16]. Because of this, it was elected to start and keep dogs on cetirizine throughout the study period; however, this change in antihistamine administration could be the cause of the increase in allergic reactions that were noted here.

Infusion reactions to ICIs occur in 3%–17% of people receiving therapy [17, 18]. Immune‐related adverse events (irAEs) due to ICIs are likely due to activation of the immune response from the very function that improves survival and the antitumour response [18]. The effect is enhanced by nonspecific T cell activation resulting in systemic inflammatory responses [19]. Allergic or infusion reactions in 16.6% of patients in the current study can likely be attributed to the drug itself and its mechanism of action.

Three dogs (17%) in this study had a Grade 3 elevation in ALP which was seen in 3.8% of patients from the manufacturer with 11.6% having a lower grade ALP elevation [8]. One theory for the increase in ALP elevations is the use of prednisone in all patients in this study, while the manufacturer safety and efficacy study did not include this. The use of prednisone caused an increase in ALP of 130% of baseline values in a study with normal beagles [20]. The incidence of liver toxicity in people receiving immune checkpoint inhibitors (ICI) is higher with combination therapy compared to monotherapy; however, this combination therapy typically refers to other ICI or tyrosine kinase inhibitors (TKI) [21]. It is possible that the combination of the therapies used in this study are a direct cause of the increase in Grade 3 metabolic changes rather than one of the therapies alone.

The NLR is a potentially valuable prognostic that is obtained with routine blood work prior to initiation of therapy. In particular, several studies have shown that an elevated NLR is reflective of an immunosuppressive phenotype and supports a negative prognosis for patients treated with immunotherapy [21, 22]. When we categorised patients into high and low NLR using the median NLR of 5.75, we noted a trend toward significance with RECIST response. With more patients, this may have ultimately resulted in significance and should be reported in future work with ICI in veterinary medicine to determine if there is an inexpensive biomarker that can predict outcomes prior to initiation of therapy that may ultimately be ineffective for a specific cohort and require substantial financial investment.

Lymph node metastasis significantly impacted both survival time and PFI. Regional lymph node involvement categorises patients into a higher stage of disease [23]. Stage has previously shown to impact outcome and, thus, should not be surprising for this cohort of patients [24]. This does raise the question of whether dogs with Stage I disease would typically be treated with systemic therapy in addition to local therapy or if the Stage I dogs were potentially overtreated in this study. Comparing this cohort to the historical data using RT and toceranib, a similar number of dogs in that study also had lymph node metastasis and appeared to outperform the dogs reported here [25]. Future studies could consider sentinel lymph node mapping and lymph node extirpation for metastatic lymph nodes to determine lymph node staging and the potential impact on tumour control and outcome with treatment with gilvetmab [26].

Immunotherapies are becoming increasingly available within veterinary oncology; however, no widely accepted, validated veterinary equivalent to immunotherapy Response Evaluation Criteria in Solid Tumours (iRECIST) exists to help recognise and identify pseudoprogression after immunotherapy [27, 28, 29]. Pseudoprogression is an increase in the size or number of lesions prior to a possibly durable response to treatment [29]. Possible pseudoprogression was reported for two dogs with melanoma treated with gilvetmab and in one dog with a glioma treated with radiation therapy in recent studies [28, 30]. Because of the limited data and reports in the literature in veterinary medicine, pseudoprogression was not considered for the eight patients who had local progression here. Validation of a veterinary‐specific iRECIST should be created to help guide veterinary oncologists on how to manage possible pseudoprogression for future patients and studies.

Limitations to this study include the small sample size, lack of histologic grading of the primary MCT and lymph nodes, possible under recognition of pseudoprogression and the lack of a control arm. Lymph node metastasis appeared to play a significant factor in outcome for both disease control and survival. The NLR might represent a biomarker for response and outcome and should be reported in future ICI work. Additionally, future studies defining the effect of RT on PD‐L1 expression in canine MCT cells may be an informative approach to help guide additional studies regarding radiation dose and timing of this combination of therapies. From the results gathered here, this sequence or combination, of therapies did not provide durable tumour control for canine MCT and the AE profile warrants further evaluation.

## Author Contributions

Conception and design: Tiffany W. Martin, Douglas H. Thamm and Kristen M. Weishaar. Acquisition of data: Tiffany W. Martin and Kristen M. Weishaar. Analysis and interpretation of data: Tiffany W. Martin, Douglas H. Thamm and Kristen M. Weishaar. Drafting the article: Tiffany W. Martin. Reviewing article for intellectual content: Tiffany W. Martin, Douglas H. Thamm and Kristen M. Weishaar. Final approval of the completed article: Tiffany W. Martin, Douglas H. Thamm and Kristen M. Weishaar. Agreement to be accountable for all aspects of the work in ensuring that questions related to the accuracy or integrity of any part of the work are appropriately investigated and resolved: Tiffany W. Martin, Douglas H. Thamm and Kristen M. Weishaar.

## Funding

This study was funded by the Dr. Larry and Ann Laiks Charitable Trust. Gilvetmab was provided by the Merck Animal Health Gilvetmab Grant Programme.

## Ethics Statement

The dogs reported in this study were client‐owned pets with naturally occurring cancer. In all cases, informed consent was obtained from the clients to treat their dogs with the reported protocol. All dogs were treated according to the principles and practises of specialty medicine prevailing at the time of their treatment.

## Conflicts of Interest

The authors declare no conflicts of interest.

## Data Availability

The data that support the findings of this study are available from the corresponding author upon reasonable request.
